# Unraveling shared molecular signatures and potential therapeutic targets linking psoriasis and acute myocardial infarction

**DOI:** 10.1038/s41598-024-67350-w

**Published:** 2024-07-16

**Authors:** Zheming Yang, Jiayin Li, Haixu Song, Zhu Mei, Shuli Zhang, Hanlin Wu, Jing liu, Chenghui Yan, Yaling Han

**Affiliations:** 1 College of Medicine and Biological Information Engineering, Northeastern University, Shenyang, 110167 Liaoning China; 2 State Key Laboratory of Frigid Zone Cardiovascular Diseases (SKLFZCD), Cardiovascular Research Institute and Department of Cardiology, General Hospital of Northern Theater Command, Shenyang, 110016 China

**Keywords:** Psoriasis, Acute myocardial infarction, Differentially expressed genes, Hub-genes, Computational biology and bioinformatics, Biomarkers, Cardiology, Molecular medicine

## Abstract

Psoriasis, a chronic inflammatory skin disorder, is associated with comorbidities such as acute myocardial infarction (AMI). However, the molecular mechanisms connecting these conditions are unclear. In this study, we conducted bioinformatics analyses using gene expression datasets to identify differentially expressed genes and hub genes associated with both psoriasis and AMI. Our findings emphasize the involvement of immune-related pathways in the pathogenesis of both conditions. Furthermore, we investigated the expression levels of hub genes in AMI patients and myocardial infarction (MI) mice. ELISA measurements revealed significantly higher levels of CXCL8, IL1B, S100A9, and S100A12 in the serum of AMI patients compared to normal individuals. Immunohistochemical staining of heart tissue from MI mice showed a progressive increase in the expression of CXCL8 and IL-1B as MI advanced, while S100A9 exhibited high expression at day 3 post-MI. mRNA expression analysis validated these findings. Additionally, we explored the skin lesions of psoriasis patients and found significantly higher expression of CXCL8, IL-1B, S100A9, and S100A12 in the affected skin areas compared to unaffected regions. These results highlight the consistent upregulation of hub genes in both AMI and psoriasis patients, as well as in myocardial infarction mice, underscoring their potential as reliable markers for disease diagnosis. Moreover, molecular docking simulations revealed potential interactions between simvastatin and key target proteins, suggesting a potential therapeutic avenue. Overall, our study uncovers shared molecular signatures and potential therapeutic targets, providing a foundation for future investigations targeting common pathways in psoriasis and AMI.

## Introduction

Psoriasis is a chronic inflammatory skin disorder that affects millions of people worldwide and has a significant impact on patients' quality of life^1,2^. It is characterized by the presence of red, scaly plaques on the skin, often accompanied by itching and pain^3^. In addition to its effects on the skin, psoriasis is now recognized as a systemic disease associated with various comorbidities, including cardiovascular diseases such as acute myocardial infarction (AMI)^4,5^.

AMI, commonly known as a heart attack, is a serious and life-threatening condition that occurs when blood flow to the heart muscle is blocked, leading to tissue damage and potential heart failure^6^. It is a major cause of morbidity and mortality worldwide^7^. Numerous epidemiological investigations have consistently shown that psoriasis significantly increases the risk of developing AMI, resulting in higher morbidity and mortality rates in affected individuals^8,9^. This correlation cannot be solely attributed to traditional cardiovascular risk factors, as the increased risk persists even after accounting for confounding variables. These observations strongly suggest the involvement of shared underlying mechanisms linking psoriasis and AMI.

**Figure 1:**
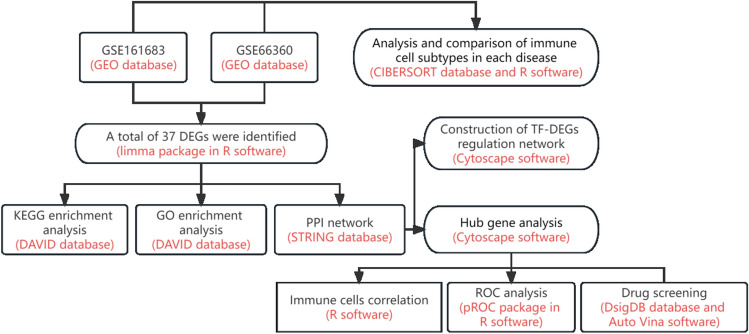
Flow chart of the analysis program in this study.

Despite the established association, the precise molecular mechanisms connecting these two conditions remain elusive. Unraveling the shared pathogenic networks between psoriasis and AMI is crucial to gain a comprehensive understanding of the complex interplay between inflammation, immune dysregulation, and cardiovascular complications. By identifying these shared pathways, we can unlock valuable insights into disease development and potentially develop targeted therapies and preventive strategies for both psoriasis and AMI. To address this research gap, we propose employing sophisticated bioinformatics analyses to elucidate the shared pathogenic networks between psoriasis and AMI. Bioinformatics techniques have proven instrumental in integrating and analyzing large-scale omics data, facilitating the identification of key molecular players and pathways involved in disease progression. By leveraging transcriptomic, proteomic, and genomic datasets from cohorts encompassing both psoriasis and AMI patients, we aim to identify common differentially expressed genes, proteins, and genetic variants that contribute to the pathogenesis of both conditions. Furthermore, network-based analysis methodologies will be employed to construct robust molecular interaction networks that integrate the identified differentially expressed molecules. These networks will allow us to explore the intricate interconnectedness and functional relationships between genes, proteins, and pathways implicated in both psoriasis and AMI. Additionally, functional enrichment analysis will be conducted to determine the biological processes, molecular functions, and cellular components enriched within the shared pathogenic networks. The specific process was shown in Fig. 1.

The findings from this study hold immense promise for shedding light on the molecular mechanisms underpinning the association between psoriasis and AMI. By unveiling the shared pathogenic networks, we can identify potential therapeutic targets that simultaneously address both conditions, paving the way for the development of innovative treatment strategies. Targeting common molecular pathways holds tremendous potential to improve patient outcomes and reduce the burden of comorbidities in individuals suffering from psoriasis and AMI. Moreover, this research carries substantial significance for personalized medicine approaches. By deciphering the molecular intricacies connecting psoriasis and AMI, we can refine risk stratification models and tailor interventions based on individualized patient profiles. This approach would enable more precise and effective management of both psoriasis-related skin symptoms and the heightened risk of AMI, ultimately improving patients' overall well-being and prognosis.

## Results

### Identification of common differentially expressed genes (DEGs) in patients with psoriasis and *AMI*

**Figure 2:**
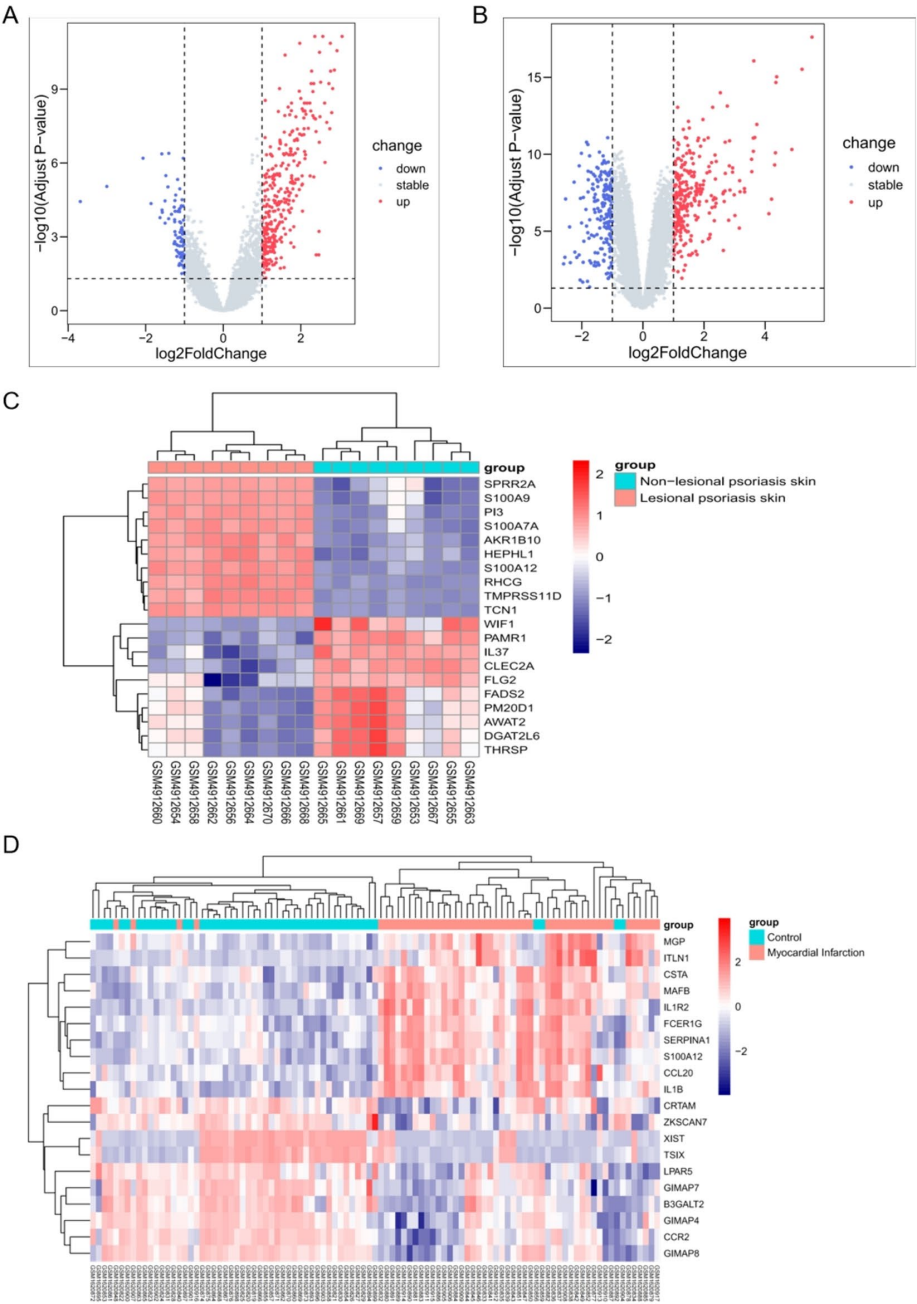
DEG identification. (**A**) Volcano plot illustrating the DEGs in GSE161683 dataset, with red indicating up-regulated genes and blue indicating downregulated genes. (**B**) Volcano plot illustrating the DEGs in GSE66360 dataset, with red indicating up-regulated genes and blue indicating downregulated genes. (**C**) Heatmaps of the top 20 DEGs showing the most significant changes in GSE161683 dataset. (**D**) Heatmaps of the top 20 DEGs showing the most significant changes in GSE66360 dataset.

In the GSE161683 dataset, a total of 194 genes showed down-regulation, 18,312 genes displayed no significant change (stable), and 287 genes exhibited up-regulation (Fig. 2A). The heat maps depict the expression patterns of the top 20 DEGs with the most significant changes (Fig. 2C). Similarly, in the GSE66360 dataset, 82 genes showed down-regulation, 20,428 genes displayed no significant change (stable), and 314 genes exhibited up-regulation (Fig. 2B). The heat maps illustrate the expression patterns of the top 20 DEGs with the most significant changes (Fig. 2D).

### Functional enrichment analysis of DEGs

**Figure 3:**
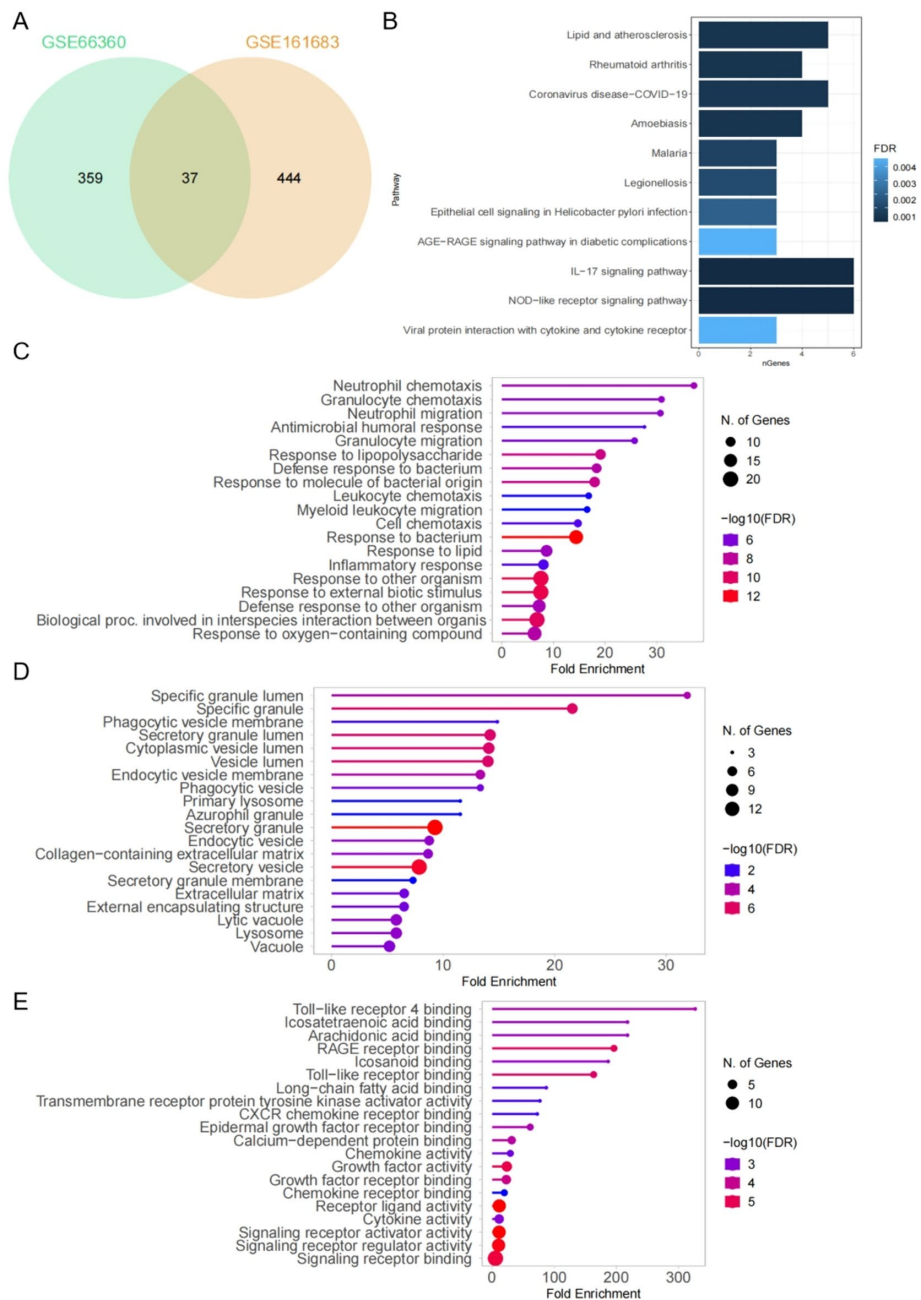
Functional enrichment analysis of DEGs. (**A**) Venn diagram depicting the overlapping DEGs between the two GEO datasets. (**B**) KEGG pathway analysis of the 37 overlapping DEGs. (**C**) GO-BP enrichment analysis of the 37 overlapping DEGs. (**D**) GO-CC enrichment analysis of the 37 overlapping DEGs. (**E**) GO-MF enrichment analysis of the 37 overlapping DEGs.

We performed functional enrichment analysis on the DEGs identified in GSE161683 and GSE66360 datasets. We found 37 overlapping genes for further investigation (Fig. 3A). In order to gain deeper insights into these DEGs, we conducted Kyoto Encyclopedia of Genes and Genomes (KEGG) pathway analysis to identify potential biological pathways involved. The analysis revealed significant enrichment of genes in the IL-17 signaling pathway and NOD-like receptor signaling pathway (Fig. 3B). Furthermore, gene ontology biological process (GO-BP) enrichment analysis showed that these proteins were primarily associated with response to external biotic stimulus, response to bacterium, and neutrophil chemotaxis (Fig. 3C). In terms of gene ontology cellular component (GO-CC), the majority of these proteins were found to be enriched in secretory granules, secretory vesicles, and specific granular cells (Fig. 3D). Lastly, gene ontology molecular function (GO-MF) enrichment analysis indicated that the 37 overlapping DEGs were related to RAGE receptor binding, Toll-like receptor binding, and signaling receptor binding (Fig. 3E).

### Analysis of protein–protein interaction (PPI) networks and transcription factor (TF) target regulating networks

**Figure 4:**
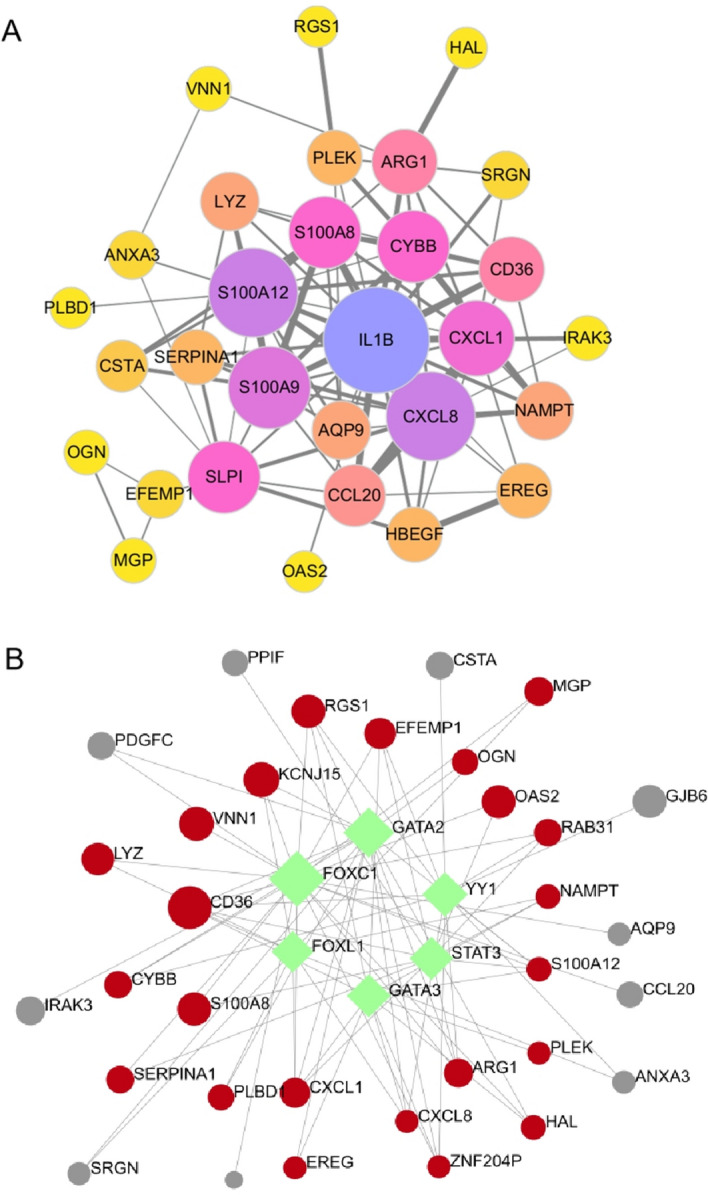
Construction of PPI and TF-DEG regulation network. (**A**) PPI network of DEGs constructed using string and Cytoscape. (**B**) TF-DEGs network consisting of 33 DEGs and 6 TFs with an NES > 4.

The PPI network of the 37 overlapping genes was constructed using the STRING database and visualized with Cytoscape software (Fig. 4A). Additionally, to gain deeper insights into the regulatory TFs and selected DEGs, we employed the iRegulon plugin to predict the TFs for the DEGs. The predicted network consisted of 33 DEGs and 6 TFs (FOXC1, GATA2, YY1, GATA3, STAT3, FOXL1). The results revealed that FOXC1 was predicted to have 21 DEG targets, GATA2 had 16 DEG targets, YY1 was associated with 12 DEGs, GATA3 was associated with 11 DEG targets, STAT3 had 11 DEG targets, and FOXL1 had 10 DEGs (Fig. 4B).

### Identification of hub genes using various ranking methods

**Figure 5:**
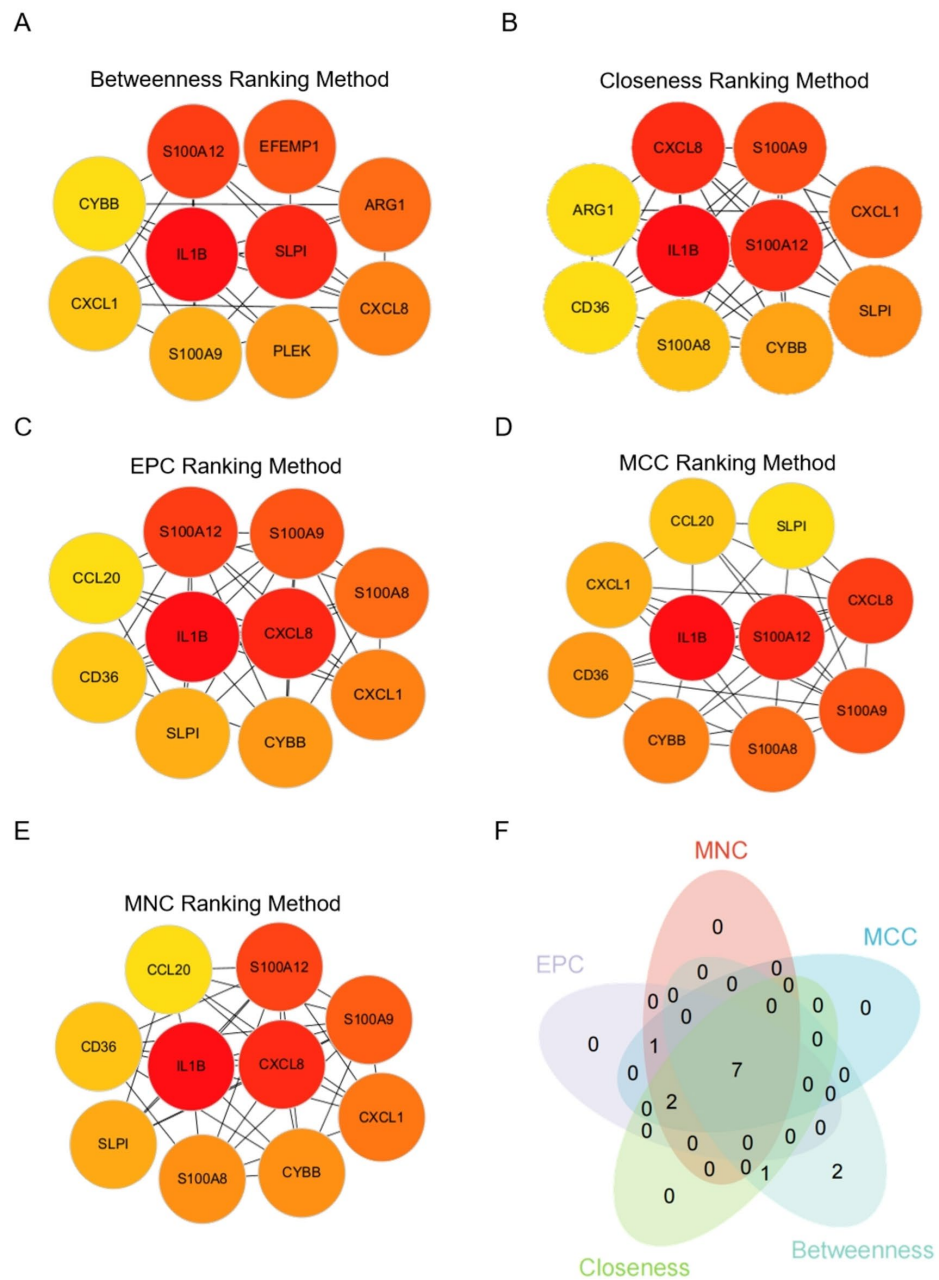
Identification of hub genes within the overlapping DEGs from the two GEO datasets. (**A**–**E**) Hub gene identification using different ranking methods: betweenness ranking, closeness ranking, edge percolated component (EPC) ranking, matthews correlation coefficient (MCC) ranking, and maximal neighborhood component (MNC) ranking. (**F**) Venn diagram illustrating the overlap of hub genes identified by different ranking methods.

Using Cytoscape v3.10.0, the query protein was visualized as a PPI network, and hub genes were identified using the cytoHubba tool, which provided a list of the top-ranked proteins. Five ranking methods were employed to determine the hub genes in CytoHubba: betweenness ranking, closeness ranking, edge percolated component (EPC) ranking, matthews correlation coefficient (MCC) ranking, and maximal neighborhood component (MNC) ranking. The top ten hub genes selected by each method were shown (Fig. 5A-E). By intersecting the results from these five approaches, SLPI, S100A9, IL1B, CYBB, CXCL8, S100A12, and CXCL1 were identified as the common hub genes involved in interactions (Fig. 5F).

## Association between psoriasis, *AMI*, and immune infiltration

**Figure 6:**
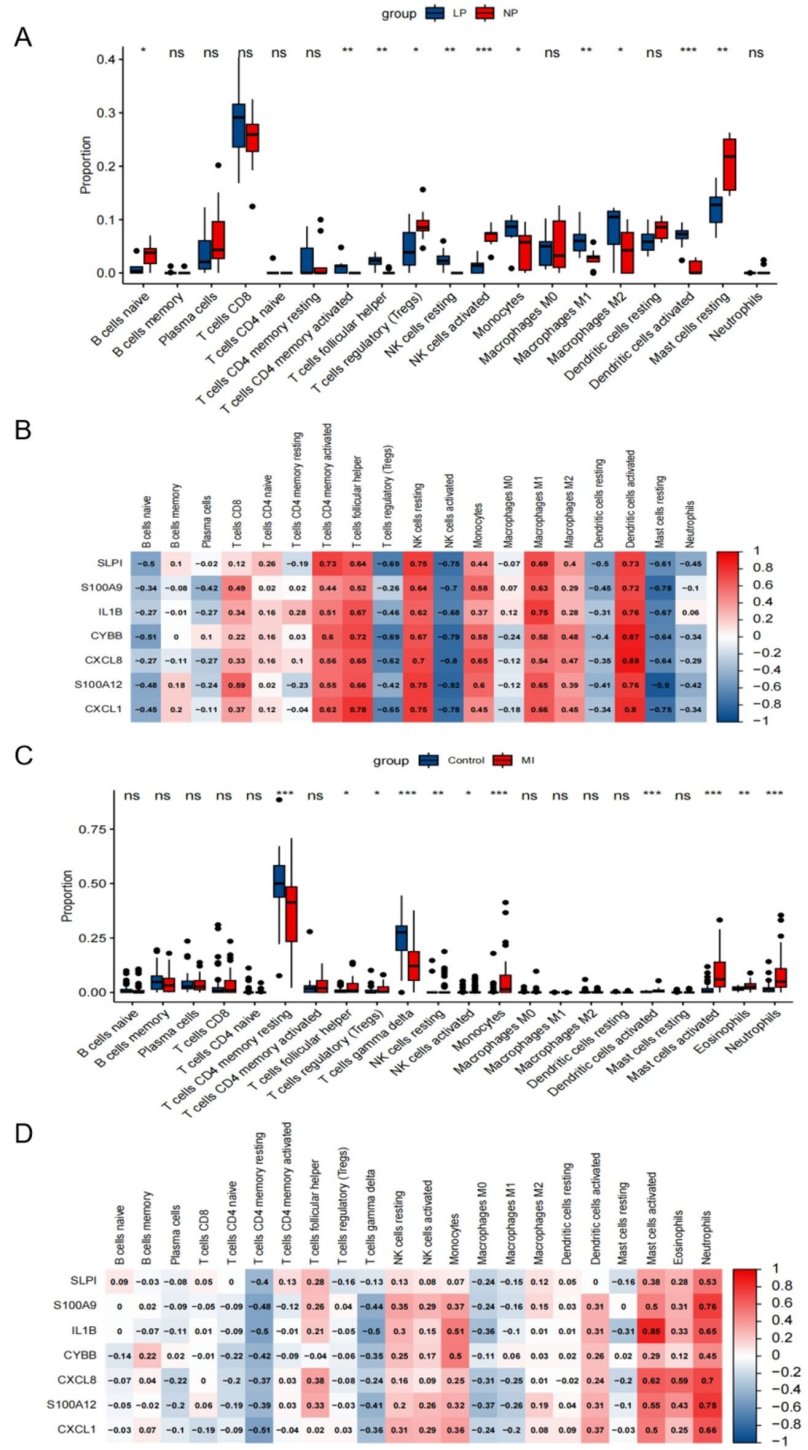
Association of immune infiltration with psoriasis and AMI. (**A**) Relative abundance of distinct immune cell subsets in GSE161683 database. (**B**) Correlation between immune cell infiltration and the expression of SLPI, S100A9, IL1B, CYBB, CXCL8, S100A12, and CXCL1 in the Psoriasis cohort. (**C**) Relative abundance of distinct immune cell subsets in GSE66360 database. (**D**) Correlation between immune cell infiltration and the expression of SLPI, S100A9, IL1B, CYBB, CXCL8, S100A12, and CXCL1 in the AMI cohort.

To explore the immunological microenvironment in psoriasis and AMI, we assessed the transcript abundance of various immune cell subtypes. Specifically, we evaluated the infiltration of immune cells in lesional (LP) and non-lesional (NP) skin of psoriasis patients. We found that the LP group had higher proportions of monocytes, M1 and M2 macrophages, activated dendritic cells, and CD4 memory resting T cells compared to the NP group (Fig. 6A). To further investigate the role of SLPI, S100A9, IL1B, CYBB, CXCL8, S100A12, and CXCL1 genes in the immune microenvironment of psoriasis, we examined inflammatory cell infiltration. Our analysis revealed significant associations of these genes with M1 macrophages and activated dendritic cells (Fig. 6B). Similarly, in the GSE66360 database, we illustrated the relative abundance of distinct immune cell subsets in the context of AMI (Fig. 6C). Compared to the Control group, the AMI group showed higher proportions of monocytes, activated dendritic cells, activated mast cells, eosinophils, and neutrophils. We also performed correlation analysis between immune cell infiltration and the expression levels of SLPI, S100A9, IL1B, CYBB, CXCL8, S100A12, and CXCL1 in the AMI cohort (Fig. 6D). These genes were significantly associated with neutrophils in the context of AMI. Notably, IL1B exhibited a significant correlation with activated mast cells.

### Expression levels and diagnostic values of hub genes

**Figure 7:**
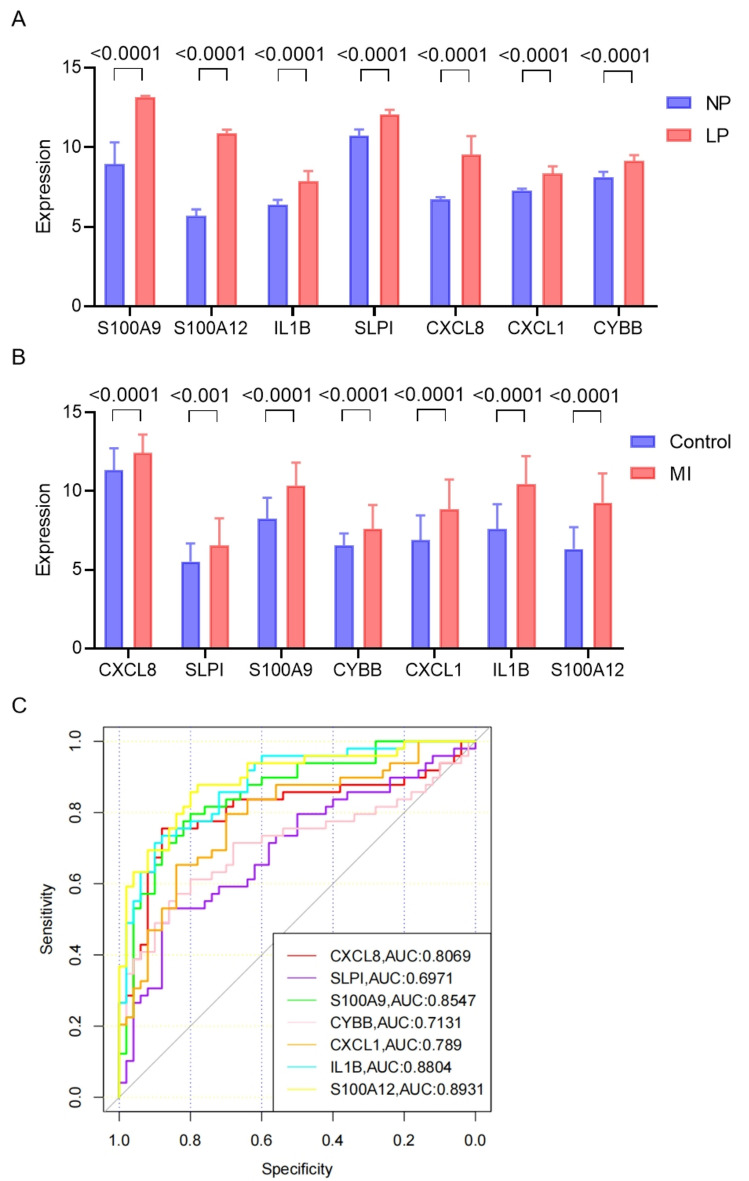
Expression levels and diagnostic values of hub genes. (**A**) Expression of hub genes in CSE161683 dataset. (**B**) Expression of hub genes in CSE66360 dataset. (**C**) ROC curve and AUC statistics of the predictive model for AMI in GSE66360 dataset.

In order to investigate the consistency of gene expression between the two diseases, we analyzed the expression of the seven differentially expressed genes in the GSE161638 and GSE66360 datasets. We observed a significant upregulation of these genes in the diseased tissues of psoriasis patients (Fig. 7A). Similarly, we found a consistent expression pattern in plasma circulating endothelial cells of patients with AMI (Fig. 7B). To evaluate the diagnostic potential of the hub genes for AMI, we developed a predictive model using the GSE66360 dataset. The predictive performance of the model was determined by the receiver operating characteristic (ROC) curve and the corresponding area under the curve (AUC) values (Fig. 7C). The ROC curve assesses the model's ability to distinguish between AMI cases and controls, while the AUC represents the overall accuracy. Among the hub genes, all except SLPI and CXCL1 exhibited AUC values greater than 0.800. Notably, S100A12 showed the highest diagnostic value with an AUC of 0.8931, followed by IL1B with an AUC of 0.8804.

### Molecular docking diagrams of simvastatin with target proteins

**Figure 8:**
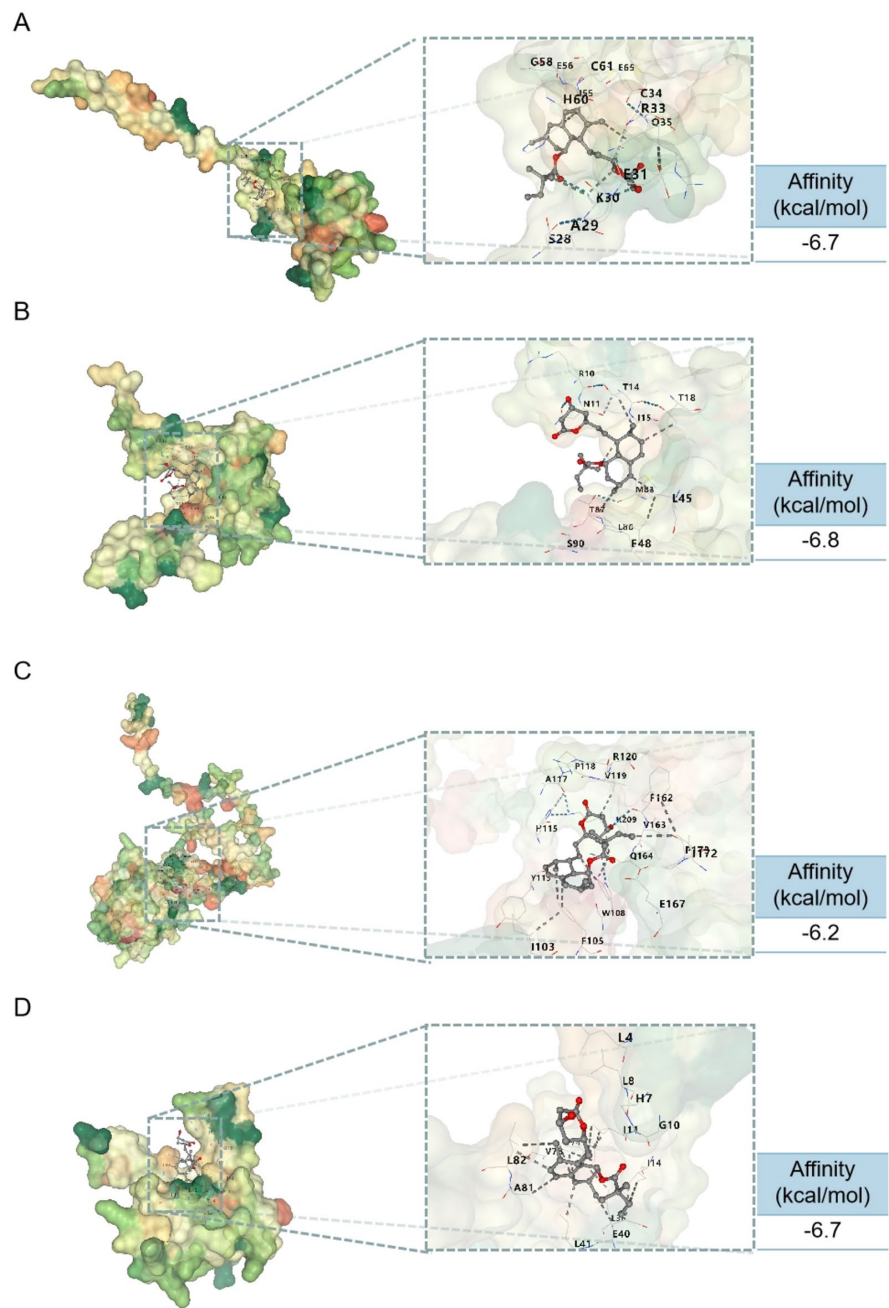
Three-dimensional molecular docking diagrams of simvastatin with target proteins: (**A**) CXCL8, (**B**) IL1B, (**C**) S100A9, and (**D**) S100A12.

To identify potential therapeutic options for psoriasis and AMI, we screened drugs targeting CXCL8, IL1B, S100A9, and S100A12 using the DsigDB database. Our findings revealed that simvastatin may be a suitable therapeutic agent for CXCL8, IL1B, and S100A9 (Supplementary Table 1). Although simvastatin was not specifically reported to interact with S100A12 in the experimentally validated database, our results demonstrated that simvastatin exhibited similar binding affinity towards S100A12 as it did towards CXCL8, IL1B, and S100A9. The molecular docking results are presented (Fig. 8A-D). These findings suggest that simvastatin could be considered as a potential therapeutic option, warranting further investigation into its mechanisms of action and therapeutic effects in the context of psoriasis and AMI.

## Hub genes are highly expressed in *AMI* and psoriasis patients as well as in myocardial infarction (MI) mice

**Figure 9:**
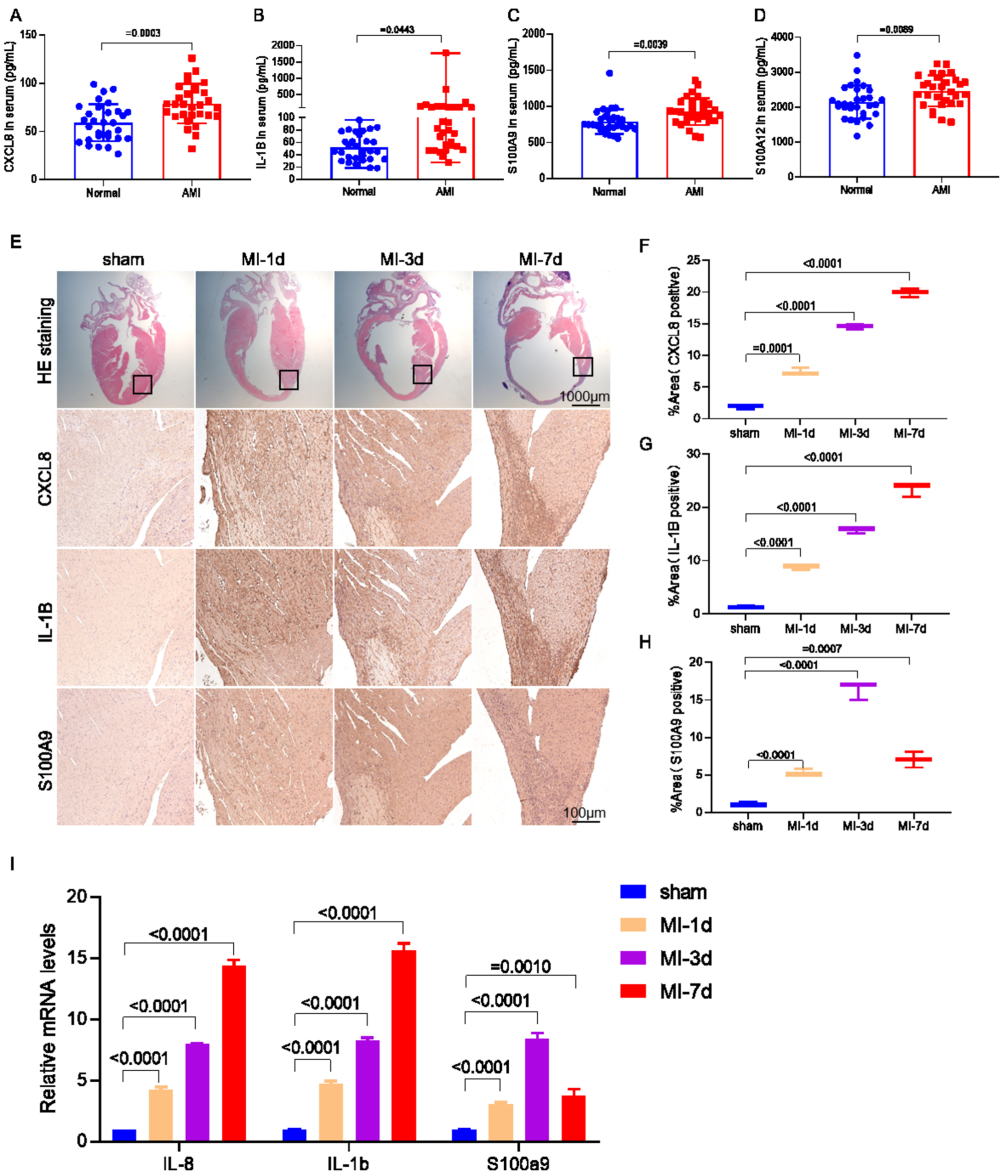
Expression of hub genes in serum of AMI patients and border zone of cardiac infarction in MI mice. ELISA was used to detect the expression of CXCL8 (**A**), IL-1B (**B**), S100A9 (**C**) and S100A12 (**D**) in serum of Normal and AMI patients. (**E**) The sham group and myocardial infarction 1 d, 3 d, 7 d heart tissue in mice of HE staining and CXCL8, IL-1B and S100A9 immunohistochemical staining and quantitative (**F**–**H**). (**I**) The mRNA expression of Il-8, Il-1b and S100a9 in cardiac tissue of MI mice on day 1, 3, 7 and sham group were detected.

In order to investigate the expression of hub genes in AMI patients, we conducted ELISA to measure the levels of CXCL8, IL1B, S100A9, and S100A12 in the serum of both normal individuals and AMI patients (Fig. 9A-D). The results clearly demonstrated that the expression of these four genes was significantly higher in patients with AMI. To further examine the impact of MI, we induced MI mice and collected heart tissue samples at 1, 3, and 7 days post-MI. Initially, we performed hematoxylin and eosin (HE) staining on the mouse heart tissue to observe the area of infarction as well as the surrounding region. Subsequently, we conducted immunohistochemical staining for CXCL8, IL-1B, and S100A9 in the edge area of the infarction. The findings revealed a progressive increase in the expression of CXCL8 and IL-1B as MI advanced, while S100A9 exhibited high expression in the heart tissues of MI mice at day 3 (Fig. 9E-H). Additionally, mRNA expression analysis of the four genes in the heart tissue of MI-induced mice confirmed the consistency with the immunohistochemical results (Fig. 9I).

**Figure 10:**
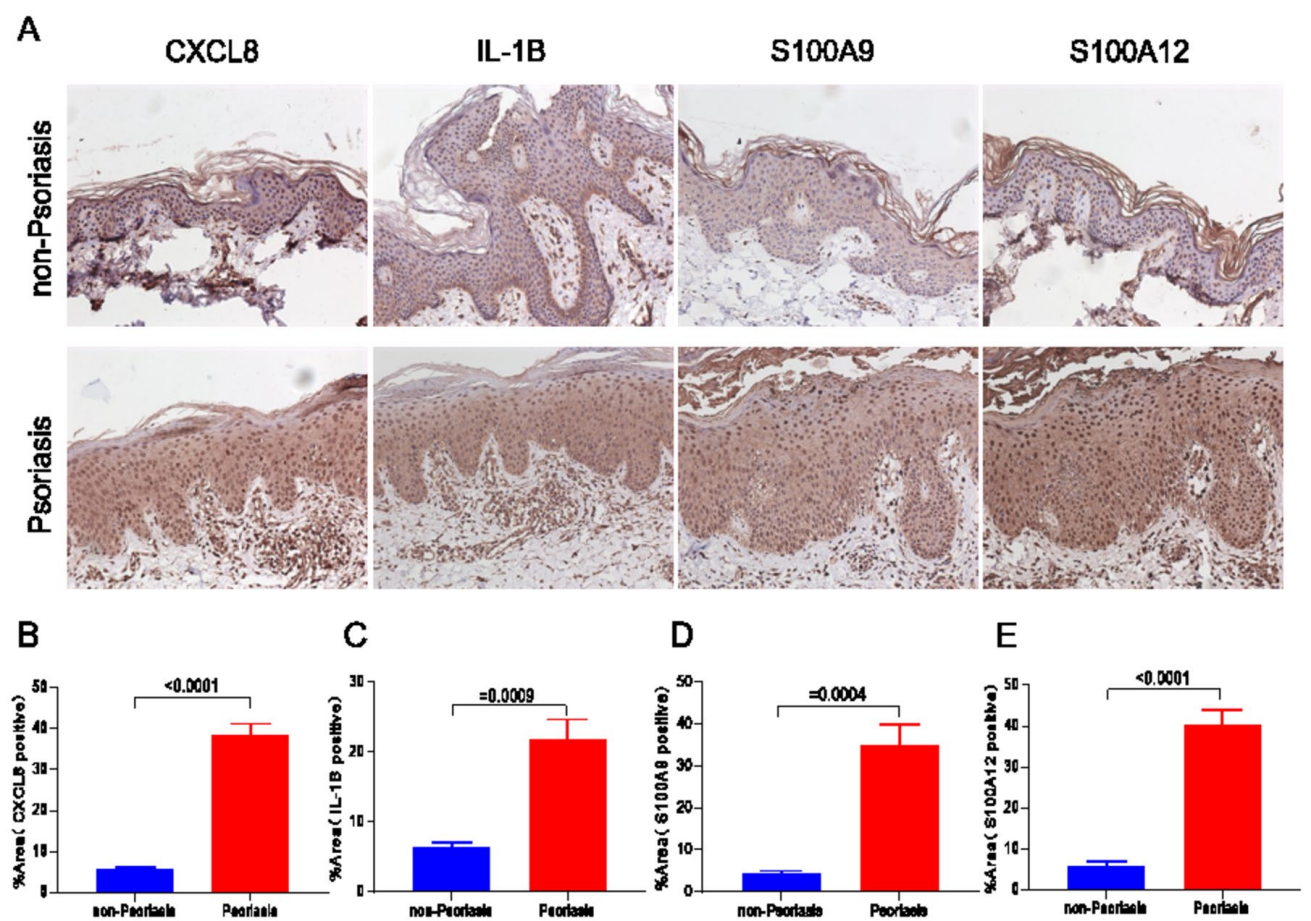
High expression of disease hub genes in psoriasis skin. (**A**) Immunohistochemical staining and quantitative (**B**-**E**) detection of the normal skin of patients with psoriasis skin disease in CXCL8, IL-1B, S100A9 and S100A12 expression.

In addition to MI, we also investigated the skin lesions of patients with psoriasis. Immunohistochemical staining of CXCL8, IL-1B, S100A9, and S100A12 in the skin lesion areas revealed significantly higher expression compared to unaffected skin regions (Fig. 10A-E).

## Discussion

In this study, we aimed to identify common DEGs in patients with psoriasis and AMI. Psoriasis is a chronic inflammatory skin disease characterized by immune dysregulation, while AMI is a cardiovascular disease associated with atherosclerosis and inflammation. Understanding the shared molecular mechanisms between these two diseases can provide valuable insights into their pathogenesis and potentially uncover novel therapeutic targets.

Our analysis of two independent datasets (GSE161683 and GSE66360) revealed a set of DEGs that showed significant changes in expression levels. Through functional enrichment analysis, we gained further insights into the biological pathways and processes involved in both psoriasis and AMI. The DEGs identified in our study were significantly enriched in the IL-17 signaling pathway and NOD-like receptor signaling pathway. These findings are consistent with previous studies that have implicated the IL-17 pathway in the pathogenesis of psoriasis^10,11^ and the NOD-like receptor signaling pathway in inflammatory responses and atherosclerosis^12,13^. The IL-17 signaling pathway plays a crucial role in the recruitment and activation of immune cells, leading to the production of pro-inflammatory cytokines and the initiation of tissue inflammation^14,15^. Activation of NOD-like receptors, on the other hand, triggers innate immune responses and the release of pro-inflammatory mediators^16^. The enrichment of DEGs in these pathways suggests that immune dysregulation and inflammation contribute to both psoriasis and AMI.

GO-BP analysis revealed that the DEGs were mainly associated with response to external biotic stimulus, response to bacterium, and neutrophil chemotaxis. This is in line with previous studies demonstrating the involvement of neutrophils in the pathogenesis of psoriasis^17,18^ and their role in the progression of atherosclerosis and AMI^19,20^. Neutrophils are key players in the inflammatory response, and their excessive activation can lead to tissue damage and disease exacerbation^21^. The enrichment of DEGs related to neutrophil chemotaxis suggests their potential contribution to the recruitment and activation of neutrophils in both psoriasis and AMI.

GO-CC analysis showed that the majority of the DEGs were enriched in secretory granules and vesicles. This finding is supported by previous studies demonstrating the involvement of secretory granules in the release of pro-inflammatory mediators, such as cytokines and chemokines, which play critical roles in the pathogenesis of psoriasis^22^ and AMI^23^. Secretory granules serve as storage sites for these bioactive molecules and their release contributes to local inflammation and immune cell recruitment^24^. The enrichment of DEGs in secretory granules highlights their potential role in regulating the secretion of pro-inflammatory mediators in both diseases.

To gain insights into the regulatory networks and TFs controlling the DEGs, we performed PPI network analysis and TF target prediction. The PPI network revealed potential interactions among the DEGs, suggesting coordinated regulation of gene expression in both diseases. The identification of hub genes using various ranking methods allowed us to prioritize the most important genes within the network. Among the hub genes, SLPI, S100A9, IL1B, CYBB, CXCL8, S100A12, and CXCL1 were consistently identified as common interacting hub genes. SLPI (Secretory Leukocyte Protease Inhibitor): SLPI is an anti-inflammatory protein that inhibits proteases and modulates immune responses^25^. SLPI is involved in regulating neutrophil chemotaxis and can contribute to tissue damage when excessively activated^26^. S100A9, also known as calgranulin B, is a calcium-binding protein expressed in myeloid cells, including neutrophils and monocytes^27^. It acts as an alarmin and plays a role in the recruitment and activation of immune cells^28^. IL1B (Interleukin-1 Beta): IL1B is a pro-inflammatory cytokine involved in the regulation of immune responses^29^. It is produced primarily by activated macrophages and plays a crucial role in the pathogenesis of various inflammatory diseases, including psoriasis and AMI^30^. CYBB (Cytochrome b-245 Beta Chain): CYBB encodes a subunit of NADPH oxidase, which is responsible for the production of reactive oxygen species (ROS) in immune cells, particularly neutrophils^31^. CXCL8 (C-X-C Motif Chemokine Ligand 8): CXCL8, also known as interleukin-8 (IL-8), is a chemokine that attracts neutrophils to sites of inflammation. It plays a critical role in the recruitment and activation of neutrophils, contributing to the inflammatory response^32^. S100A12: S100A12, also known as calgranulin C, is another member of the S100 protein family. It is predominantly expressed in neutrophils and monocytes and functions as an alarmin in inflammation^33^. S100A12 has been implicated in various inflammatory conditions, including psoriasis and cardiovascular diseases^34^. CXCL1 (C-X-C Motif Chemokine Ligand 1): CXCL1, also known as growth-regulated oncogene-alpha (GRO-α), is a chemokine involved in the recruitment of neutrophils and monocytes to inflamed tissues^35^. These genes have been implicated in inflammation, immune responses, and disease pathogenesis, supporting their potential roles as key regulators in both psoriasis and AMI.

We observed distinct patterns of immune cell infiltration in the lesional and non-lesional skin of psoriasis patients, as well as in the context of AMI. Psoriasis is characterized by immune cell infiltration, primarily driven by activated T cells and dendritic cells^36^. Our findings support these observations and demonstrate an increased proportion of monocytes, M1 macrophages, M2 macrophages, activated dendritic cells, and CD4 memory resting T cells in the lesional skin of psoriasis patients. These immune cell subsets play critical roles in the pathogenesis of psoriasis through the production of inflammatory cytokines and the activation of adaptive immune responses. Similarly, AMI is associated with immune cell infiltration in the affected cardiac tissue^37^. We observed an increased proportion of monocytes, activated dendritic cells, activated mast cells, eosinophils, and neutrophils in the context of AMI. These immune cell subsets contribute to the inflammatory response and tissue damage following AMI. The correlation analysis between the hub genes and immune cell infiltration highlighted their potential roles in modulating specific immune cell types. For example, SLPI, S100A9, IL1B, CYBB, CXCL8, S100A12, and CXCL1 were found to be highly associated with M1 macrophages, activated dendritic cells, and neutrophils. These findings suggest that the hub genes may contribute to the recruitment and activation of these immune cell subsets, further promoting inflammation in both psoriasis and AMI.

To assess the diagnostic potential of the hub genes, we evaluated their expression levels in the diseased tissues of psoriasis patients and plasma circulating endothelial cells of AMI patients. Consistent upregulation of these hub genes was observed in both diseases, indicating their potential as reliable markers for diagnosing psoriasis and AMI. Furthermore, the diagnostic model developed using the hub genes exhibited promising performance, with high AUC values for most of the hub genes. Notably, S100A12 showed the highest diagnostic value, followed by IL1B. These findings suggest that the hub genes could serve as potential diagnostic markers for distinguishing psoriasis and AMI cases from controls. This is consistent with previous studies reporting the diagnostic value of these genes in psoriasis^38,39^ and AMI^40,41^.

To explore potential therapeutic options, we selected four proteins with an AUC > 0.800 for further analysis. We predicted potential therapeutic agents that could target these proteins and discovered that simvastatin could be a promising treatment option for both psoriasis and AMI. Simvastatin is a widely used statin medication known for its lipid-lowering effects^42^. Beyond its lipid-lowering properties, simvastatin has demonstrated anti-inflammatory and immunomodulatory activities^43^. Through molecular docking analysis, we identified potential binding interactions between simvastatin and CXCL8, IL1B, S100A9, and S100A12. These findings suggest that simvastatin may have therapeutic effects in mitigating the inflammatory processes associated with both psoriasis and AMI.

While our study provides valuable insights into the shared molecular mechanisms between psoriasis and AMI, there are certain limitations that need to be acknowledged. Firstly, our findings are based on the analysis of publicly available gene expression datasets. Although we utilized two independent datasets to increase the robustness of our results, the heterogeneity of these datasets and potential batch effects may introduce bias and affect the generalizability of our findings. Secondly, the sample sizes in these datasets were relatively small. Although these datasets provided valuable insights into the molecular mechanisms underlying psoriasis and AMI, the limited sample sizes may introduce statistical uncertainties and reduce the power of our analyses. Hence, replication studies with larger cohorts are necessary to enhance the robustness and generalizability of our findings. Finally, although the potential therapeutic effects of simvastatin on target proteins have been investigated through molecular docking analysis, further in vitro and in vivo studies are necessary to validate these interactions and evaluate the effectiveness and safety of simvastatin as a potential treatment for psoriasis and AMI.

In conclusion, our study identified common DEGs, functional pathways, and regulatory networks associated with both psoriasis and AMI. The overlapping genes enriched in immune-related pathways and their association with immune cell infiltration highlight the importance of immune dysregulation in the pathogenesis of these diseases. Moreover, the hub genes identified in this study provide valuable insights into the central players and potential diagnostic markers for psoriasis and AMI. Further investigation of these DEGs and hub genes may contribute to a better understanding of the shared molecular mechanisms underlying psoriasis and AMI, ultimately leading to the development of novel therapeutic strategies for managing these diseases.

## Methods

### Ethics

GEO is public database. The patients listed in the database have ethical clearance. Users can submit pertinent articles and get pertinent data for free. Sera were obtained from normal subjects (n = 30) as well as patients admitted to hospital with a diagnosis of AMI (n = 30), aged between 40 and 60. Skin tissue from patients with psoriasis was obtained from patients diagnosed with psoriasis, and normal tissue was obtained from portions of undiseased tissue from the same patient. All samples were collected following The Code of Ethics of the World Medical Association and with the informed consent of the patients/donors.

### Animals

Male C57BL/6 mice aged 8 weeks were purchased from Shanghai Model Organisms Center (shanghai, China), and raised at the specific pathogen-free (SPF) laboratory animal facility. Mice were maintained on a 12-h light/dark cycle at 25 °C and provided free access to commercial rodent chow (sterilized by Cobalt-60) and tap water (high-temperature sterilization) before initiation of the experiments. Randomized grouping was used and the same group of mice were co-housed with less than 5 animals per cage. All animal experiments were complied with Directive 2010/63/EU, Commission Implementing Decision (EU) 2020/569, Recommendation 2007/526/EC and 1991 International Guidelines for Ethical Review of Epidemiological Studies.

Left anterior descending coronary artery was ligated to create the MI mice model. Under sterile conditions, left anterior descending coronary artery was tied by a 7–0 silk suture. Sham-operated (Sham) mice were treated with the same surgery without tying the left anterior descending coronary artery.

### Immunohistochemical staining

Skin tissues obtained from human psoriasis patients and heart tissues collected from mice with myocardial infarction were fixed in 4% paraformaldehyde for 48 h. Subsequently, the tissues were dehydrated, embedded in paraffin, and sectioned to prepare histological samples for observation. Immunohistochemical staining was performed on the tissue sections using an immunohistochemical kit (Maxim Biology, China, KIT-9730). Antibody Application: Anti-CXCL8 antibody (Proteintech, 27095-1-AP, dilution ratio: 1:100); Anti-IL-1B antibody (Affinity, DF6251, dilution ratio: 1:100); Anti-S100A9 antibody (ABclonal, A22131, dilution ratio: 1:100); Anti-S100A12 antibody (ABclonal, A12499, dilution ratio: 1:100).

### ELISA

ELISA was employed to detect the levels of CXCL8, IL1B, S100A9, and S100A12 in serum samples obtained from acute myocardial infarction (AMI) patients (n = 20) and AMI mice (n = 6). The ELISA kits used in this study were procured from Animaluni (China) under the product codes LV10281, LV10309, LV10651, LV10435, LV30300, LV30328, and LV30461.

### RNA isolation and real-time PCR

RNA extraction was performed using TRIzol reagent (Invitrogen). Using a PrimeScriptTM RT reagent Kit (Takara, Beijing, China) with gDNA Eraser, 1 g of total RNA was reverse-transcribed into cDNA. On a CFX96 TouchTM Real-Time PCR Detection System (Hercules, CA, USA), quantitative real-time PCR was conducted using TB Green® Fast quantitative polymerase chain reaction (qPCR) Mix (Takara) and specific primers (Ribobio).

### Data source and DEG identification

We utilized the GEO database to obtain two datasets, namely GSE161683 for psoriasis and GSE66360 for AMI. The GSE161683 dataset provided gene expression profiles of LP and NP psoriasis skin samples from 9 patients, using microarray analysis. Similarly, the GSE66360 dataset included information on circulating endothelial cells from patients with AMI (n = 49) as well as healthy control subjects (n = 50). Both male and female patients were included in these datasets. To analyze the differential gene expression, we performed standardization and differential analysis using R Project. Specifically, we employed LIMMA and DESeq2 packages for data standardization and differential analysis. Volcano plots and heatmaps were generated using R to visualize the significantly DEGs. The statistical significance cutoff for DEGs was set at |log2(FC)|> 1 and *p* < 0.05.

### KEGG and GO enrichment analysis

To conduct pathway enrichment analysis, we utilized the DAVID database for KEGG pathway enrichment^44^ and GO enrichment^45^ analysis of the 37 DEGs. We selected the top 11 pathways for KEGG studies and the top 20 for GO studies based on their biological relevance. To identify significantly enriched pathways, a false discovery rate (FDR) value of less than 0.05 was applied as the cutoff criterion.

### Construction of PPI and TF-DEG regulation network

To integrate biomolecular interaction networks, high-throughput expression data, and other molecular states, we obtained protein–protein and functional interaction networks for the 37 combined hub genes from the STRING database (https://string-db.org/). These networks were visualized using Cytoscape^46^. To identify potential interactions between DEGs and TFs, we utilized the iRegulon plugin in Cytoscape. This plugin helped identify TFs that potentially target the DEGs. Enriched motifs in iRegulon were ranked based on direct targets using position weight matrix 50. The TF-DEG crosstalk pairs were obtained from databases such as TRANSFAC, JASPAR, and others. Finally, the TF-DEG regulatory network was visualized using Cytoscape.

### Identification of hub genes

To explore the hub gene network, we utilized the CytoHubb Plugin in Cytoscape. This plugin provides various techniques for identifying critical nodes within biological networks and their interactions with other genes. In our study, we employed multiple algorithms (including betweenness, closeness, EPC, MCC, and MNC ranking methods) to identify the top 10 hub genes. The resulting network was colored to reflect the relevance of the hub nodes; red indicating the highest score and yellow indicating the lowest. By performing a Venn diagram analysis on the top 10 hub genes from each ranking method, we identified SLPI, S100A9, IL1B, CYBB, CXCL8, S100A12, and CXCL1 as the common interacting hub genes.

### Immune cell infiltration and correlation analysis

To investigate the immune microenvironment in psoriasis and AMI patients, we analyzed the immune cell infiltrations using the CIBERSORT algorithm^47^. This algorithm allowed us to determine the proportions of different immune cell types present. We depicted the expression levels of 22 immune infiltrating cells using box plots. To visualize the relationship between different immune cell types and SLPI, S100A9, IL1B, CYBB, CXCL8, S100A12, and CXCL1, we used the ggplot2 package and performed Spearman correlation analysis.

### Identification of diagnostic genes

To determine the predictive values of the hub genes, we conducted ROC curve analysis and calculated the corresponding AUCs using the pROC package in R software. Diagnostic genes were selected from GSE66360 dataset based on the criterion of having an AUC greater than 0.800. To observe the dynamic changes in the diagnostic genes in AMI patients, we analyzed the ROC curves of these diagnostic genes in both AMI and control patients.

### Prediction of potential therapeutic drugs

We utilized the DSigDB database^48^ to predict potential therapeutic drugs targeting the hub genes. The chemical structure of simvastatin was obtained from a reliable source and converted into a suitable format compatible with the molecular docking software. The ligand was then prepared by adding hydrogen atoms and optimizing its geometry. Crystal structures of the target proteins (CXCL8, IL1B, S100A9, and S100A12) were retrieved from the PDB database. The protein structures were prepared by removing any existing water molecules, heteroatoms, and other ligands. Molecular docking simulations were performed using the AutoDock Vina program^49^. The results of the molecular docking simulations were visualized using PyMOL software, generating three-dimensional docking diagrams that depict the predicted binding modes of simvastatin within the binding sites of each target protein (CXCL8, IL1B, S100A9, and S100A12).

### Statistical analysis

We utilized R 4.2.0 software and GraphPad Prism (version 8.0.1) for data analysis in this research. Data are presented as the mean ± SD, and comparisons between groups were performed using an unpaired Student's *t* test (LP vs NP, AMI vs Control). ROC curves were used to evaluate the AUC and the predictive abilities of the models. A p-value less than 0.05 was considered statistically significant.

### Supplementary Information

Supplementary Information.

## Data Availability

The data used to support our results are available at the GEO (https://www.ncbi.nlm.nih.gov/geo/query/acc.cgi?acc=GSE66360; https://www.ncbi.nlm.nih.gov/geo/query/acc.cgi?acc=GSE161683)**.**

## Abbreviations

AMI: Acute myocardial infarction
NASH: Nonalcoholic steatohepatitis
HCC: Hepatocellular carcinoma
DEGs: Differentially expressed genes
KEGG: Kyoto encyclopedia of genes and genomes
GO-BP: Gene ontology biological process
GO-CC: Gene ontology cellular component
GO-MF: Gene ontology molecular function
PPI: Protein–protein interaction
TF: Transcription factor
ROC: Receiver operating characteristic
AUC: Area under the curve
